# Surface-Based Cortical Measures in Multimodal Association Brain Regions Predict Chess Expertise

**DOI:** 10.3390/brainsci12111592

**Published:** 2022-11-21

**Authors:** Nicolò Trevisan, Assia Jaillard, Giulia Cattarinussi, Prisca De Roni, Fabio Sambataro

**Affiliations:** 1 Department of Neuroscience (DNS), University of Padova, 35122 Padova, Italy; 2 Padova Neuroscience Center, University of Padova, 35122 Padova, Italy; 3 AGEIS, Université Grenoble Alpes (UGA), 38058 Grenoble, France; 4 IRM 3T Recherche, IRMaGe, Centre Hospitalier Universitaire Grenoble Alpes (CHUGA), 38043 Grenoble, France; 5 Department of Developmental Psychology and Socialization (DPSS), University of Padova, 35122 Padova, Italy

**Keywords:** gyrification, fractal dimension, cortical complexity, structural magnetic resonance imaging, chess expertise

## Abstract

The complex structure of the brain supports high-order cognition, which is crucial for mastering chess. Surface-based measures, including the fractional dimension (FD) and gyrification index (GI), may be more sensitive in detecting cortical changes relative to volumetric indexes. For this reason, structural magnetic resonance imaging data from 29 chess experts and 29 novice participants were analyzed using the CAT12 toolbox. FD and GI for each brain region were compared between the groups. A multivariate model was used to identify surface-based brain measures that can predict chess expertise. In chess experts, FD is increased in the left frontal operculum (*p* < 0.01), and this change correlates with the starting age of chess practice (ρ = −0.54, *p* < 0.01). FD is decreased in the right superior parietal lobule (*p* < 0.01). Chess expertise is predicted by the FD in a network of fronto-parieto-temporal regions and is associated with GI changes in the middle cingulate gyrus (*p* < 0.01) and the superior temporal sulcus (*p* < 0.01). Our findings add to the evidence that chess expertise is based on the complex properties of the brain surface of a network of transmodal association areas important for flexible high-level cognitive functions. Interestingly, these changes are associated with long-lasting practice, suggesting that neuroplastic effects develop over time.

## 1. Introduction

The game of chess is a complex intellectual activity that provides a useful model for the study of memory, attention, perception, visuospatial cognition, and problem-solving [1]. Success in chess appears to be related to several factors, including experience in chess playing, participation in tournaments, fluid intelligence, spatial processing, and social cognition [2,3,4]. A good performance in chess is related to intensive practice over the years, with a minimum of ten years of practice required at the grandmaster level [5]. Cognitive factors may also contribute to chess expertise. A meta-analysis of cognitive abilities in chess players showed that chess skills are positively correlated with fluid intelligence, comprehension, general knowledge, working memory, and processing speed [6].

Morphological neuroimaging studies explored how structural measures contribute to determining the neural correlates of chess expertise. Voxel-based morphometry (VBM) studies reported structural differences by comparing brain cortical and subcortical structures between chess players and novices with little or no experience in the chess game. Overall, chess players show decreased gray matter (GM) volume and cortical thickness (CT) in the caudate nucleus [7], the frontal and parietal gyri [8], and the occipitotemporal junction, along with increased mean diffusivity in the left superior longitudinal fasciculus (SLF) [9]. A recent study reports thinner CT in expert chess players compared to novices in the bilateral frontoparietal regions [8]. Moreover, greater expertise is correlated with decreased mean diffusivity in the right SLF [9], and the duration of professional training and cognitive scores are associated with diffusion measures in the association white matter tracts, including the uncinate fasciculus, inferior longitudinal (ILF), and SLF [10]. Taken together, these studies suggest that chess expertise is associated with structural changes in a distributed network of regions engaged in cognitive tasks related to intelligence and visuospatial abilities with rather low regional specificity.

Brain volumetric approaches (e.g., VBM) are robust methods that have been extensively used for the study of neurophysiological processes, as well as for neuropsychiatric disorders. Unfortunately, these methods may be inaccurate during spatial normalization to a standard template [11] for registration errors and may produce inflated statistics [12] (see Goto et al. [13], for a more detailed comparison of the two approaches). Complementary to this approach, surface-based morphometry was introduced to provide more accurate information on cortical changes relative to VBM [11]. Indeed, surface-based morphometry can measure different properties of the cortical surface, including the gyrification index (GI) and the fractal dimension (FD). In particular, GI is defined as the ratio of the pial surface area to the surface area of the cortical hull or the outer contour of the brain [14]. GI in a large set of associated regions is associated with general cognitive ability and intelligence, accounting for 5–12% of the variance in general intelligence [14,15]. FD is a nonlinear measure derived from fractal geometry that quantifies self-similarity, a measure that outperforms traditional Euclidean geometrics for the description of irregular surfaces. FD can be defined as an index of complexity that assesses how a detail in a fractal pattern changes with a varying measuring scale. Since the highly convoluted brain cortex represents a fractal structure [16,17,18,19,20,21], FD has been used to describe the morphology of the brain cortical surface and to assess the cortical complexity at the level of the brain hemispheres, regions, and neurons [22,23,24,25].

Neuroimaging studies show that FD correlates with both morphological complexity and neuronal maturity [17]. Furthermore, recent work in the literature shows that FD is associated with fluid intelligence, particularly information processing, and the ability to generate, test, and refute multiple hypotheses simultaneously [26]. Therefore, FD appears to be closely related to working memory, attention, and visuospatial processing [15], which are crucial skills in the expertise of chess.

Gyrification is considered a potential marker of early neurodevelopment since the formation of gyri and sulci in the brain begins between 10 and 15 weeks of human fetal life and reaches its peak during the third trimester of fetal life [27]. Conversely, FD increases from fetal life through childhood and into adulthood, until it starts to decrease later in life [27,28,29]. Interestingly, both GI and FD provide useful information on cognitive abilities due to their close relationship with innate intelligence and with developmental changes, respectively [15,16]. Furthermore, decreased FD has also been observed in several neuropsychiatric conditions associated with altered cognitive function [30,31].

In this study, our objective was to investigate changes in the brain surface of chess experts using GI and FD. We hypothesized that the surface indexes of the brain regions and networks underlying high-order cognition, including fluid intelligence, working memory, processing speed, and visuospatial processing, namely, prefronto-parieto-temporal networks, would be altered. Furthermore, since chess training usually starts during childhood, we hypothesized that these indexes would be correlated with the age of chess practice.

## 2. Materials and Methods

### 2.1. Participants

We used data extracted from the Huaxi MR Research Center database [32]. This database contains healthy participants’ data from 29 professional chess players (age: 28.72 ± 10.84 years; 9 females) and 29 novices (age: 25.76 ± 6.95 years; 15 females) with very limited skills and knowledge of the chess game. Professional chess players received rigorous training (training time: 4.24 ± 1.73 h/day). They started playing at 8.50 ± 2.80 years old and professional training at 17.00 ± 5.80 years old, respectively. The professional chess players had an average score of 2401.1 ± 134.6 Elo chess skills. Specifically, 6 of them were rated grandmasters and 11 masters. Additionally, 23 of these professional chess players scored above the entry level for chess mastery by the standards of the United States Chess Federation. The two groups were matched for age, sex, and education (Table 1). All participants were right-handed and had no history of physical or mental disorders.

### 2.2. Brain Imaging

#### 2.2.1. Data Acquisition

A high-resolution T1-weighted structural image was acquired for each subject, using an MPRAGE sequence. The scanning parameters were the following: TR = 1900  ms, TE = 2.26  ms, TI = 900  ms, bandwidth = 200  Hz/Px, FOV = 256 × 256  mm^2^, flip angle = 9°, 176 slices, voxel size = 1 × 1 × 1 mm^3^.

#### 2.2.2. Pre-Processing

**Figure 1:**
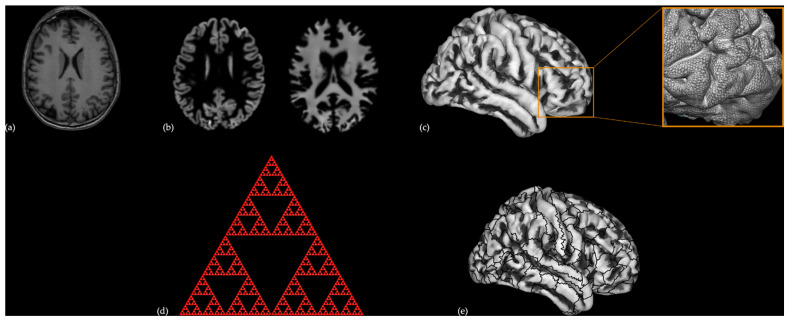
Surface-based cortical measures [fractal dimension (FD) and gyrification index (GI)] estimation process. (**a**) Structural T1 image (in coronal orientation) was registered, normalized, and segmented to extract the; (**b**) grey matter (left) and white matter (right) components. From these maps (**c**) the cortical mesh was reconstructed using the projection-based thickness method (the inset illustrates an example of cortical mesh in the right prefrontal cortex); (**d**) the FD (in the figure, a Sierpiński triangle, which exemplifies the self-similarity concept, i.e., the large equilateral triangle can be decomposed into smaller equilateral triangles at different scales, is depicted) and the GI (not represented) were calculated from the cortical mesh; (**e**) the cortical surface was parcellated in 180 regions of interest (ROI) per hemisphere using the Human Connectome Project multi-modal parcellation atlas and FD and GI values were averaged within each ROI.

T1 images (Figure 1a) were spatially registered to the Montreal Neurological Institute (MNI) template, and then segmented into GM, white matter (WM), and cerebrospinal fluid (CSF) components (Figure 1b) using DARTEL [33]. Segmented data were used to reconstruct the cortical surface of each participant (Figure 1c). The central surface reconstruction included topology correction, spherical inflation, and spherical registration. The central surface was used as an input to calculate GI and FD (Figure 1d). These values were analyzed following the specifics reported by Dahnke et al. [34], using the approach of “spherical harmonic reconstructions” proposed by Yotter and colleagues [23]. Finally, the mean values of FD, GI, and CT were extracted for 180 regions of interest (ROI) for each hemisphere (Figure 1e) as defined in the Human Connectome Project (HCP) multi-modal parcellation atlas [35]. All analyses were performed using the Computational Anatomy Toolbox for SPM (CAT12, http://www.neuro.uni-jena.de/cat/ (accessed on 16 December 2020)).

To confirm our approach, we wanted to replicate the findings of Ouellette and colleagues on GI differences in professional chess masters in the same dataset using a different software (CAT12 vs. Freesurfer) and analysis approach (high-resolution ROI vs. whole brain) [8] (see Supplementary Materials).

### 2.3. Statistical Analysis

#### 2.3.1. Bivariate Analysis

Demographic data were tested for normality using the Shapiro-Wilk test. Bivariate comparisons were performed using chi-square tests for categorical variables and with two-sample *t*-tests or Mann–Whitney tests for continuous variables according to their distribution, with a false discovery rate (FDR) correction for multiple comparisons, respectively. The bivariate correlations between GI and FD and the behavioral variables provided in the dataset were carried out using Pearson’s or Spearman’s correlation tests according to the distribution of the variables. The correlations of variables related to the starting age of the chess training of the expert players were controlled by the age, sex, and education of the participants. The level of significance was set to *p* < 0.05 for all tests. We also performed an outlier analysis on the GI and FD values using the Grubbs’ method and found no outliers in the data. Additionally, we performed vertex-wise whole brain two-sample *t*-tests between the two player groups, where the FD, GI, and CT measures for both hemispheres were merged and resampled to a 32 k mesh resolution, with a 25 mm smoothing with a full width half maximum Gaussian kernel (FWHM). We then used threshold-free cluster enhancement (http://www.neuro.uni-jena.de/tfce (accessed on 16 December 2020)) with 5000 permutations and applied a family-wise-error (FWE) corrected threshold of *p* < 0.05 to control for multiple comparisons.

#### 2.3.2. Multivariate Analysis

To estimate the association of regional surface-based values with chess expertise, we performed logistic regression (LR) analyses, controlling for the effects of age, sex, and education. Two LR models (one for FD and another for GI) were estimated with chess skills as a dependent variable. Covariates were introduced in the model using block entry for demographic variables and a forward conditional stepwise method for regional GI and FD. The regions were pre-selected based on regions that reached significance in bivariate analyses comparing GI and FD in professional chess experts and novices at a threshold of *p* < 0.05 uncorrected. We evaluated the performance of the LR model using the chi-square likelihood ratio test, statistical tests of individual predictors (betas) using the Wald chi-square statistic and *p* < 0.05, goodness-of-fit using the Hosmer and Lemeshow test, and the Nagelkerke pseudo-R^2^ index, and predicted probabilities using a classification table assessing model accuracy. Internal validation was applied to correct for overfitting with bootstrap based on 5000 replications. Finally, we determined the regions in which FD and GI were associated with chess expertise by assessing the sensitivity and specificity of the LR models using the receiver operator characteristic (ROC) providing an area under the curve (AUC) estimate, with the highest AUC considered as indicating the best model (see Supplementary Materials). Statistical analyses were performed using Jamovi software (Version 1.2, https://www.jamovi.org, (accessed on 16 December 2020)), RStudio (http://www.rstudio.com/, (accessed on 16 December 2020)), and SPSS (IBM SPSS Statistics for Windows, Version 20.0, IBM, Armonk, NY, USA).

## 3. Results

### 3.1. Cortical Complexity Assessed by FD

#### 3.1.1. Bivariate Comparison

Compared to novices, professional chess players show significantly higher FD values in the left frontal operculum OP5 (lFOP5, *p* = 0.030) and the precentral operculum (PrCO), and significantly lower FD values in the right area 7M (7 m, *p* = 0.030), after FDR correction for multiple comparisons.

**Figure 2:**
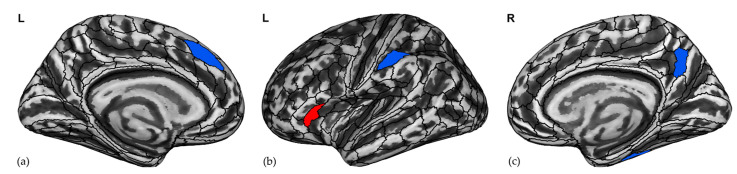
Increased (in red) cortical complexity in the (**b**) left frontal operculum OP5 (left FOP5) and a decrease (in blue) in (**a**) the left caudal part of the dorsomedial prefrontal cortex (8BM), (**b**) the left inferior parietal lobule with a thin cortical ribbon (PFt) and (**c**) the right 7 m and right temporal area F (TF), respectively, predict chess expertise using logistic regression. L, left; R, right.

From the additional whole-brain vertex-wise analysis, professional chess players show a cluster of significantly higher FD in an area located in the left frontal operculum (x, y, z = −34, 28, 13, k = 12, *p* = 0.010, FWE-corrected) (Figure 2).

#### 3.1.2. Correlations with Chess-Related Features in Chess Masters

**Figure 3:**
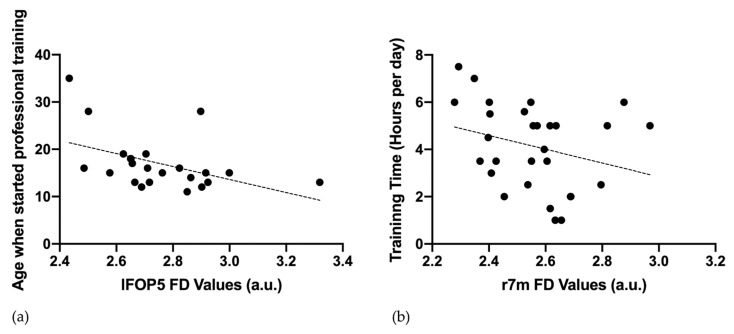
Scatter plots of fractal dimension (FD) values and demographic and behavioral data among professional chess players. (**a**) The FD in the left frontal operculum OP5 (lFOP5) is correlated with the age (years) at which the participants begin their professional training (ρ = −0.503, *p* = 0.008). (**b**) The FD in the right area 7M (r7m) is correlated with the daily time spent in chess training (ρ = −0.403, *p* = 0.034). a.u. = arbitrary units.

Increased FD in lFOP5 is inversely correlated with the starting age of professional chess training (ρ = −0.544, *p* = 0.007) (Figure 3a). Furthermore, reduced FD on the right 7 m of professional chess players shows a trend for negative correlation with the daily duration of chess training (ρ = −0.384, *p* = 0.040) (Figure 3b). No significant correlation is found between the FD and Elo scores.

### 3.2. Regions Associated with Chess Expertise

The LR model controlling for age, sex, and education (Table 2) shows that chess expertise is predicted by increasing FD in the left FOP5 and decreasing FD in the right 7 m, right temporal area F (TF), left caudal part of the dorsomedial prefrontal cortex (8BM), and part of the inferior parietal lobule with a thin cortical ribbon (PFt). Chess expertise is associated with FD values in a set of association regions including the left fronto-opercular cortex, the right SPL/posterior cingulum and the lateral temporal cortex, and the fronto-medial and IPL cortices (Figure 2). Moreover, younger age is significantly associated with chess expertise, with no significant effect of sex or education. The efficiency of the model reaches 93.1%, the Nagelkerke R^2^ = 0.793, and the AUC = 0.961 (SE = 0.025, 95%CI = 0.912 to 1.000), indicating high model performance. A full list of ROIs with significantly different FD values in professional chess players compared to novice participants using two-sample *t*-tests is reported in Table S1.

### 3.3. Gyrification Index

**Figure 4:**
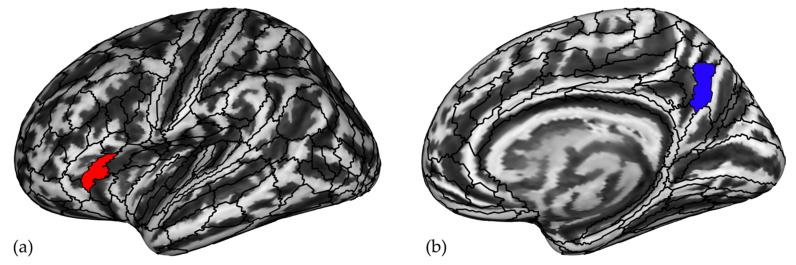
Increased gyrification index in (**a**) the posterior part of the right anterior cingulate cortex (a24pr, in red) and decreased in (**b**) the superior and posterior part of the right superior temporal sulcus (STSdp, in blue) predicted chess expertise using logistic regression. R, right.

We do not find significant differences between the GI of professional chess players and novices after correcting for multiple comparisons. Given that the GI distribution is normal in all ROIs, we performed two-sample *t*-tests showing significant differences in 11 ROIs listed in Table S2. These ROIs were introduced into the LR model after controlling for the effects of age, sex, and education. The resulting LR model shows that GI is predicted by two ROIs, the posterior part of the right anterior cingulate cortex (24 prime, a24pr) and the superior and posterior part of the right superior temporal sulcus (STSdp and STSpr), as reported in Figure 4 and Table 3. The accuracy of the model is good [efficiency = 69%, AUC = 0.798 (SE = 0.058, 95% CI = 0.685 to 0.911)].

### 3.4. Cortical Thickness

We find no differences between chess-player groups when correcting for multiple comparisons. Chess experts present a reduction in CT mainly in the left visual (V6), premotor (pre-SMA), and paralimbic (OFC) areas and in a set of regions that span the bilateral frontal, temporoparietal, and visual cortices using an uncorrected threshold of *p* < 0.05 (see Table S3 for detailed results).

## 4. Discussion

This study investigated differences in surface-based cortical measures assessed with FD and GI in whole brain areas parcellated using the HCP atlas in 29 chess experts and 29 novices. We find that experts show an increase in FD in the left FOP5, which is correlated with the starting age of chess training, and a decrease in FD in the right SPL-7 m area, with a trend for a negative correlation between FD and the duration of daily training. When applying a logistic regression model, FD predicts chess expertise in a network of transmodal association regions, including the SPL-7 m and lateral temporal cortex in the right hemisphere and the fronto-opercular cortex FOP5, fronto-medial cortex 8BM, and IPL-PFt in the left hemisphere. Age, but not gender and education, have a significant effect on the model. Regarding GI, we find no significant differences between the two groups, after correction for multiple comparisons. Nevertheless, when using GI values from ROIs with significant differences in experts, chess expertise is predicted by two areas: the posterior part of the right anterior cingulate cortex and the posterior part of the right STSdp.

We find that chess experts have increased FD in the left FOP5, this region lies in the precentral part of the frontal operculum, rostrally to BA44. Although the role of FOP5 has not been fully explored, the frontal operculum is an important component of the attentional and memory circuits. For instance, this region is more active in professional musicians when simulating or imagining playing a well-known piece (retrieval of motor memory) [36]. FOP5 is also implicated in visuomotor learning, the selection of competing alternatives, and the retrieval and maintenance of rules, specifically when they are related to the context or when the subject is required to keep in mind a set of rule contingencies [37,38,39]. Moreover, increased performance in the executive component of a task is correlated with increased activation of the lateral frontal operculum [40] and can contribute to the transition from default mode to a task-positive network [41]. More recently, FOP5 has been identified as a region of the extended multi-demand cognitive network, implicated more in relational tasks than in math and working memory tasks [42]. This is the first time that neural changes in this region are associated with chess expertise. FOP5 may be involved in various complex cognitive tasks, including the maintenance of multimodal mental representations that can promote high cognitive efficiency in chess experts. In particular, the level of player expertise, measured by the age of starting chess training, is positively correlated with the left FOP5 FD. These findings are in line with the previous literature that reveal that intensive training and learning processes produce changes in neurogenesis, glial genesis, and remodeling of different cellular and vascular components of the brain, resulting in regional structural and functional reorganization [43], especially from childhood to adulthood [44]. The higher complexity in the left frontal operculum could reflect an increased processing effort in this area during chess playing. Since the frontal operculum is a phylogenetically old area that underlies several complex multimodal cognitive processes, when challenged, it cannot hyperspecialize. Thus, to maximize its efficiency, its complexity is increased.

Area 7 m is a heteromodal associative region located in the superior parietal lobule and the precuneus [35]. Specifically, the superior parietal lobule is known to mediate several functions related to spatial processing, such as spatial attention, remapping of attentional priorities, and mental rotation [45,46,47,48]. It also plays an important role in the integration of visual and motor information, which is important for visually guided actions [49]. Moreover, area 7m is involved in the manipulation of information in working memory [50]. Thus, SPL could be involved in chess skills for its role in spatial visual processing, spatial attention, and working memory [49]. Area 7m plays a central role in a variety of integrated tasks, such as visuospatial imagery, memorization, and temporal processing of multiple task timelines [51,52], and in mentalization and cognition. Moreover, the right precuneus is involved in controlling the spatial aspects of motor behavior [53,54]. Interestingly, as previously reported by Ouellette and colleagues, chess experts have cortical thinning in the left SPL and precuneus [8]. Our study also shows a trend for an inverse correlation between cortical complexity in 7m and the duration of chess training. This suggests that starting chess training in the middle of childhood may have enhanced cortical thinning, and this may lead to greater cognitive efficiency [9]. Taken together, our findings suggest that a reduction in cortical complexity in the SPL and precuneus could be associated with better chess skills and performance, due to intensive cognitive training.

PFt is an association area located in the anterior part of the inferior parietal lobule that is mainly connected to the sensorimotor circuit. The PFt area has been reported to be a key node of a network that aims to generate purposeful hand actions in human and non-human primates [55]. In particular, the left PFt is also considered a part of the so-called salience network [56], directing attention to the most important stimuli in the environment. Interestingly, two studies also implicate IPL functional connectivity in chess experts. Both studies find a greater connectivity of IPL with the visuomotor network [57] and the frontoparietal network [58] in chess experts that are correlated with the duration of professional chess playing. Consistent with our findings in area r7m, the reduced FD could reflect a higher specialization of this area after intensive training, producing a faster and more efficient processing of the chess moves.

Area TF is a limbic area of the lateral parahippocampal cortex located in the ventromedial part of the inferior temporal gyrus. TF is part of a large association network of regions including STS, visual area V4, and retrosplenial cortex, as well as multimodal association regions of the prefrontal, insular, cingulate, and posterior parietal cortices. Given its highly interconnected nature, area TF is highly engaged in tasks involving spatial information about the environment and, in particular, the processing of contextual associations [59] that support chess expertise through the holistic processing of various classes of stimuli. This is in line with an fMRI study showing that chess experts have increased activity in the collateral sulcus and the bordering area TF when looking at chess boards with plausible game positions, compared to boards where pieces were placed randomly [60]. Consistently, a higher level of chess expertise is correlated with diffusion MRI connectometry in the bilateral ILF [10], an association white matter tract connecting the parahippocampal and extra-striate occipital cortices.

Area 8BM is the caudal part of the human dorsomedial prefrontal cortex that belongs to the core multi-demand cognitive network of regions. This network is activated by a broad domain of tasks integrating brain processing to access and bind information and cognition operations required for the complex behaviors that are required for chess playing [42].

Overall, chess expertise appears to be associated with cortical complexity changes in various regions engaged by tasks such as spatial information processing, mathematics, conceptualization, and social cognition. These findings suggest that the neural substrates involved in chess expertise can be defined within the broader framework of a network connecting transmodal and paralimbic association regions of the prefrontal, opercular, cingulate, dorsomedial parietal, and temporal cortices. In this view, FD appears to be a useful measure for a quantitative description of the structural complexity of the brain cortex, particularly in transmodal regions with flexible and high-level social and cognitive functions that are not well captured by CT measures. FD condenses cortex details into a single numeric value, which is an extremely compact measure of cortical complexity.

In particular, previous studies exploring FD in neurological disorders point toward the idea that a decrease in FD is associated with brain damage, yielding gray matter [30,61,62,63,64], and few investigations show an increase of this measure in pathological conditions [30]. Our findings of decreased FD in chess experts in right 7 m, right PF, left 8 bm, and left PFt suggest that reductions in FD in healthy subjects are not related to gray or white matter lesions, but can result from long-term and intense training that yields the refinement of association neural circuits via selective synaptogenesis and synaptic pruning.

Chess expertise is predicted by an increase in GI in the anterior part of the middle cingulate cortex and a decrease in GI in the superior and posterior part of the STS [45]. These are two key regions of the theory of mind network [61,62], which is responsible for the mental representation of others’ intentions and expectations in social interactions, including the prediction of deceptive behaviors [63] and the comprehension of the intentions of actions [62]. Specifically, STS is involved in social cognition and motor skills [61] and the cingulate cortex in mentalizing functions and in the response to the selection of cognitive output [64], respectively. GI changes in these areas can reflect the ability to understand the implications of the position of each piece in the game and to decode the emotions of the opponent, select the appropriate response, and take advantage of it in the match strategy. We do not find an effect of chess training on GI. This is expected, as GI changes are reported to occur during pregnancy and the first years of infancy and to remain relatively stable throughout life [65].

Cortical complexity assessed with FD is altered in chess experts in two regions implicated in high-level cognitive tasks and modulated by chess training. Our findings extend the reports of altered CT in chess experts [8] to a more complex measure that can reflect the characteristics of chess training. In particular, chess expertise is associated with differences in FD in a set of transmodal association late-maturing regions that undergo structural and functional changes until early adulthood [44]. Indeed, FD, unlike GI, is likely to change in response to cognitive demands throughout life [25,26,27,30,65], and its changes can contribute to the performance of complex cognitive behaviors, including chess expertise. However, more research is needed to assess whether FD can be a reliable method to investigate cortical changes related to expertise.

Lastly, we also investigated CT in chess players and novices and found widespread cortical thinning in frontoparietal and visual areas involving primary, unimodal, and heteromodal areas. Our results replicate the findings reported by Ouellette and colleagues [8]. Of note, Ouellette et al. [8] investigated CT in the same dataset but using a different processing tool, Freesurfer versus CAT12, and a different cortical parcellation atlas, indicating that our results were not influenced by the processing steps that were used to estimate CT.

Furthermore, a series of studies investigated the neural correlates of chess expertise using the same dataset, although focusing on resting-state connectivity and brain volumetry. In one study that investigates the dynamic resting state functional connectivity, the chess masters show enhanced global dynamic fluidity, operating over an extended dynamic range [66]. Another resting state study shows increased functional connectivity between the posterior fusiform gyrus and the visuospatial attention and motor networks in chess players [57]. A surface-based study of cortical thickness by Ouellette and colleagues reports cortical thinning in professional chess players in the left SPL and precuneus [8]. Finally, only a VBM study is performed in this dataset and finds a significant reduction in the thalamic volume in chess masters, and this volume is correlated with the level of chess expertise and the training time [58]. In our study, we analyze the complexity of cortical folding, a surface-based index, which is complementary to other morphometric approaches in the identification of structural changes in the cortex [30]. In particular, cortical complexity summarizes information from different gray matter components, thus, resulting in a greater ability to detect changes associated with brain aging, cognition, and neuropsychiatric disorders, including those associated with cognitive impairment compared to the traditional surface- and volume-based approaches [30]. Thus, our findings extend previous results of structural changes in the thalamus and parietal cortex to a broader structural network, including prefronto-tempo-parietal regions, which is consistent with the functional results indicating increased overall brain dynamics and functional connectivity in parieto-temporal networks, which may underlie the spatial information processing, working memory, conceptualization, and mentalization that can be necessary to become a chess master.

Some limitations must be acknowledged. The sample size of this study is small. However, the study is sufficiently powered to identify group differences for morphometric measures, as confirmed by previous studies on the same database [32]. Another limitation is the type of phenotypic characterization of the participants that is limited to measures of demographic and chess expertise, which is sufficient for the purpose of the study, but hinders the possibility of investigating specific cognitive skills. Future studies incorporating thorough neurocognitive measures are needed.

## 5. Conclusions

This study investigated the neural basis of chess expertise by exploring the differences in FD and GI in chess experts and novice participants. We show that chess expertise is associated with FD changes in a flexible and interactive network of transmodal areas that integrate visuospatial information, working memory, abstraction, mentalization, and social cognition functions that promote the development of high-level skills and confer advantages to chess experts over novices. These findings add to previous evidence that the neural bases of chess expertise are related to a network of transmodal regions with a functional organization influenced by a variety of developmental, structural, and environmental factors [44]. This study also emphasizes that brain processes can be explored using cortical complexity assessed with FD. Future studies with larger sample sizes and more detailed cognitive information will allow a better and more in-depth understanding of the neural substrates of chess playing. We also suggest that studying the brain structure of chess players longitudinally would shed light on the nature of FD changes and their ontogenetic processes. Future research should also investigate the effects of extensive cognitive training on brain structure, not only due to chess play but also due to the learning of complex cognitive skills. Furthermore, longitudinal studies aimed at identifying brain areas that mediate complex cognitive skills in learning, including mastering chess, could be useful for a better understanding of learning and for the use of neurostimulation techniques to improve this process.

## Figures and Tables

**Table 1:** Demographic and chess training characteristics of professional chess players (N = 29) and novice participants (N = 29).

	Professional Chess Players (N = 29)	Novices (N = 29)	*p*-Values *
Age: mean (SD)	28.72 (10.84)	25.76 (6.95)	0.22
Sex: females (%)	9 (31.03%)	15 (51.72%)	0.11
Education: years (SD)	13.27 (2.79)	13.92 (3.15)	0.41
Elo rank: mean (SD)	2401.1 (134.6)	-	-
Age at which they started professional training: mean years (SD)	17 (5.8)	-	-
Duration of daily training: mean hours (SD)	4.12 (1.79)	-	-

SD = Standard deviation; * Mann–Whitney test.

**Table 2:** Logistic regression model predicting chess expertise based on the fractional dimension of the specific regions of interest (ROI), after controlling for age, sex, and education.

Predictors with Bootstrap						
	B	Bias	SE	*p*	95% CI	
					Lower	Upper
Age (years)	−0.10	−5.98	34.31	0.025	−52.27	1.27
Sex (male)	1.01	61.15	561.57	0.317	−135.33	595.34
Education	−0.24	−13.81	107.35	0.100	−118.85	16.58
*ROIs*						
Left FOP5	11.07	485.79	2869.57	0.001	5.94	3844.66
Left PFt	−7.25	−412.05	2380.84	0.000	−3538.41	−4.18
Left 8BM	−16.41	−698.42	3863.81	0.001	−5819.85	−9.42
Right TF	−8.92	−404.94	2514.75	0.004	−3164.28	−3.45
Right 7 m	−7.67	−214.67	1686.45	0.002	−1616.48	63.18
Intercept	74.24	3258.26	18,208.48	0.000	51.04	26,353.63
**Classification table**						
	Predicted					
Observed	Novices		Professional chess players	Correct %		
Novices	26		3	89.7%		
Professional chess masters	1		28	96.6%		
Overall percentage				93.1%		
**Model fit**	Hosmer and Lemeshow test					
Nagelkerke R^2^			Chi-2	p		
0.793			5.724	0.678		

The significance of the factors (*p*-values) and 95% confidence intervals (95% CI) of the B values are indicated based on 5000 bootstrap samples. SE, standard error; 8BM, caudal part of the dorsomedial prefrontal cortex; FOP5, frontal operculum OP5; PFt, inferior parietal lobule with a thin cortical ribbon; TF, temporal area F.

**Table 3:** Logistic regression model predicting chess expertise based on the gyrification index of the specific regions of interest (ROI), after controlling for age, education, and sex.

Predictors with Bootstrap						
Predictors	B	Bias	SE	*p*	95% CI	
					Lower	Upper
Education	1.04	0.19	0.95	0.106	−0.27	3.00
Age (years)	0.04	0.01	0.07	0.367	−0.05	0.18
Male/female	−0.10	−0.01	0.19	0.441	−0.48	0.24
ROIs						
Right a24pr	−0.37	−0.08	0.26	0.007	−0.95	−0.09
Right STSdp	0.55	0.14	0.44	0.007	0.20	1.46
Intercept	−5.64	−2.13	13.46	0.503	−34.00	11.46
	**Classification table**					
		Predicted				
Observed	Novices	Professional chess masters		Correct %		
Novices	21	8		72.4%		
Professional chess masters	10	19		65.5%		
Overall percentage				69.0%		
**Model fit**	Hosmer and Lemeshow test					
Nagelkerke R2		Chi-2		p		
0.359		7.030		0.533		

The significance of the factors (*p*-values) and 95% confidence intervals (95% CI) of the B values are indicated based on 5000 bootstrap samples. SE, standard error; a24pr, posterior part of the right anterior cingulate cortex; STSdp, superior and posterior part of the right superior temporal sulcus.

## Data Availability

Public archiving of individual data is available at http://fcon_1000.projects.nitrc.org/indi/pro/wchsu_li_index.html, accessed on 16 December 2020.

## Supplementary Materials

The following supporting information can be downloaded at: https://www.mdpi.com/article/10.3390/brainsci12111592/s1, Table S1. List of Region Of Interest (ROI) with significant different FD values in chess expert compared to novice participants using two-sample t-tests (p values uncorrected for multiple comparisons). Table S2. List of Region Of Interest (ROI) with significant differences in GI values in participants with chess expertise compared to novice participants using two-sample t-tests (p values uncorrected for multiple comparisons). ROIs in bold are predictors in the logistic regression model. Table S3: Cortical thickness comparisons between Chess experts (n = 29) and Novices participants (n = 29) showing decreased cortical thickness in most brain regions using Mann-Whitney test with p values uncorrected for multiple comparisons.
