# Supplementary material for: Surface-Based Cortical Measures in Multimodal Association Brain Regions Predict Chess Expertise
Source: Brain Sci. 2022 Nov 21;12(11):1592. doi: 10.3390/brainsci12111592 (PMC9688322; doi:10.3390/brainsci12111592)
Supplement: Supplementary file 1 [file brainsci-12-01592-s001.zip › brainsci-1948430-supplementary.pdf]

Table S1. Regions of interest (ROI) with significant different fractal dimension values in professional chess players compared to novices using two-sample t-tests (p values uncorrected for multiple comparisons). ROIs in bold are predictors in the logistic regression model.

ROIs	t	p (2-tailed)	ROI Location
**Left FOP5**	4.19	<0.001	Frontal Operculum (FOP)
**Right 7m**	-4.03	<0.001	Medial SPL / Posterior Cingulate cortex (PCC)
**Right TF**	-3.21	0.002	Lateral temporal cortex
Right FOP5	2.91	0.005	Frontal Operculum (FOP)
Right STGa	-2.79	0.007	Superior temporal gyrus (Auditory associative cortex)
**Left PFt**	-2.78	0.007	Inferior Parietal Lobule (PFt Area)
Left AVI	-2.74	0.008	Anterior Ventral Insular Area (AVI)
Left FOP4	2.66	0.010	Frontal Operculum (located ventrally to FOP5)
Right PF	2.49	0.016	Inferior Parietal Lobule (PF Area)
Left 31a	2.45	0.017	Dorsal Cingulate cortex
Left IFJp	-2.41	0.019	Inferior frontal cortex (IFJ)
Left TF	-2.39	0.020	Ventromedial part of the inferior temporal gyrus (parahippocampal region)
Right FST	2.39	0.020	Fundus of the Superior Temporal visual areas
Right 52	2.31	0.025	Transitional Auditory area-Insula
Left p32	2.29	0.026	Pregenual Anterior Cingulate cortex
Left V6A	2.19	0.032	Occipital cortex (posterior SPOC)
Left RSC	-2.15	0.036	Retrosplenial cortex (RSC)
Left 4	-2.15	0.036	Primary Motor cortex
Right PHA2	2.15	0.036	ParaHippocampal (area 2)
Right 31pd	-2.12	0.038	Posterior Cingulate cortex
Left PHA2	2.12	0.039	ParaHippocampal (area 2)
Right DVT	2.07	0.043	dorsal visual transitional area (PCC)
Left SCEF	2.07	0.043	Suppl. Cingulate Eye Field (dorsomedial PFC)
Right 24dd	-2.06	0.044	Mid Cingulate cortex
**Left 8BM**	-2.05	0.045	dorsomedial PFC
Left FOP1	2.02	0.048	Frontal Operculum (FOP anterior part)

SPL indicates Superior Parietal Lobule, PFC prefrontal cortex and PCC posterior cingulate cortex

Table S2. Regions of interest (ROI) with significant differences in gyrification index values in professional chess masters compared to novices using two-sample t-tests (p values uncorrected for multiple comparisons). ROIs in bold are predictors in the logistic regression model.

ROIs	t	p value	ROI Location
**Right a24pr**	-2.41	0.019	Cingulate cortex
**Right STSdp**	2.40	0.020	Posterior STSd
Left 3a	-2.39	0.021	Somatosensory cortex
Right IFJa	-2.33	0.024	Inferior frontal gyrus
Right TF	2.25	0.028	Inferior temporal gyrus
Right H	2.18	0.034	Hippocampus
Right LIPd	2.16	0.035	SPL (medial bank of IPS)
Right A5	2.08	0.042	Auditory 5 Complex
Left LIPv	-2.08	0.042	SPL (medial bank of IPS)
Left EC	2.07	0.043	Entorhinal (temporal cortex)
Left 8Ad	2.01	0.049	Dorsolateral PFC

SPL indicates Superior Parietal Lobule, PFC prefrontal cortex, PCC posterior cingulate cortex and IPS intraparietal sulcus

Table S3
Cortical thickness comparisons between professional chess players (n=29) and novices (n=29) showing decreased cortical thickness in most brain regions using Mann-Whitney test with p values uncorrected for multiple comparisons.

Anatomical region	HCP Atlas ROI	statistics (Mann-Whitney Z-scores)	p-value
Dorsolateral prefrontal cortex	Right SFL	-2.75	0.006
Motor cortex	Left 4	-2.40	0.016
Premotor cortex	Right 55b	-2.42	0.016
Dorsolateral prefrontal cortex	Left 9p	-2.08	0.038
Dorsal cingulate cortex	Right 24dd	-3.36	0.001
Dorsolateral prefrontal cortex	Right 8C	-2.89	0.004
	Right p9-46v	-2.37	0.018
Supplementary and Cingulate Eye Field	Right SCEF	-2.25	0.025
Premotor cortex	Right 6mp	-2.17	0.030
Supramarginal	Right PFt	-3.15	0.002
	Right PFm	-3.09	0.002
	Right IP2	-2.15	0.032
	Right PGp	-2.08	0.037
Visual cortex	Left V6	-3.69	0.000
	Left V2	-3.19	0.001
	Left V3A	-3.35	0.001
	Left V3CD	-2.79	0.005
	Left VVC	-2.75	0.006
	Right V1	-2.46	0.014
	Left V4	-2.39	0.017
	Left V3	-2.30	0.021

	Left V1	-2.20	0.028
	Right V3A	-2.19	0.029
	Left V3B	-2.03	0.042
Frontal Premotor preSMA	Left 6mp	-3.93	0.000
Frontal Premotor SMA	Left 6ma	-3.38	0.001
Dorsolateral prefrontal cortex	Left 8C	-2.75	0.006
	Right 9a	-2.61	0.009
Medial prefrontal cortex	Left 8BM	-2.57	0.010
Frontal Premotor cortex	Left 6a	-2.20	0.028
	Left 55b	-2.09	0.036
Superior Parietal	Left 7PC	-2.87	0.004
	Left 7PL	-2.42	0.016
	Left 5mv	-2.25	0.025
	Left 7Am	-2.25	0.025
Inferior Parietal	Left PFop	-3.00	0.003
Supramarginal	Left PGi	-2.95	0.003
ventral lateral intraparietal area	Left LIPv	-2.90	0.004
Inferior Parietal	Left PFm	-2.74	0.006
superior temporal gyrus region a	Right STGa	-2.59	0.010
Inferior Parietal	Left PFcm	-2.20	0.028
Parietal Sensory	Left 3b	-3.03	0.002
Parietal Sensory	Left 2	-3.06	0.002
Anterior midcingulate cortex	Left a24pr	-3.10	0.002
Parietal Sensory	Left 1	-2.87	0.004
Superior Parietal	Left 5m	-2.62	0.009
Superior Parietal	Left 5L	-2.56	0.011
Parietal Sensory	Left 3a	-2.25	0.025

Orbital and polar frontal cortex	Left OFC	-3.55	0.000
	Left 10d	-3.04	0.002
	Left a47r	-2.76	0.006
	Right 11l	-2.68	0.007
Entorhinal Cortex	Left EC	-2.53	0.012
PeriSylvian Language Area	Left PSL	-2.36	0.018
Orbital and polar frontal cortex	Left pOFC	-2.34	0.019
Precuneus and Superior Parietal	Left VIP	-2.99	0.003
	Left 7AL	-2.71	0.007
	Left LIPd	-2.70	0.007
	Right 7PC	-2.62	0.009
	Left MIP	-2.60	0.009

Cortical thickness methods

Cortical thickness (CT) values were estimated in one step using a projection-based distance measure (Dahnke et al., 2013). From these ROIs, we have selected the 60 regions of the HCP atlas that occupied the same space of the regions that Ouelette and colleagues reported to have significantly lower cortical thickness in chess players when compared to controls. Due to the distribution of the variables, we performed Mann–Whitney test for each region, and obtained the same results as those reported by Oulette and colleagues.
